# Development of a Theoretically Driven mHealth Text Messaging Application for Sustaining Recent Weight Loss

**DOI:** 10.2196/mhealth.2343

**Published:** 2013-05-07

**Authors:** Ryan J Shaw, Hayden B Bosworth, Jeffrey C Hess, Susan G Silva, Isaac M Lipkus, Linda L Davis, Constance M Johnson

**Affiliations:** ^1^School of NursingDuke UniversityDurham, NCUnited States; ^2^Center for Health Services ResearchDurham Veterans Affairs Medical CenterDurham, NCUnited States; ^3^Department of MedicineDuke University Medical CenterDurham, NCUnited States

**Keywords:** mHealth, short message service, SMS, text messaging, weight loss maintenance

## Abstract

**Background:**

Mobile phone short message service (SMS) text messaging, has the potential to serve as an intervention medium to promote sustainability of weight loss that can be easily and affordably used by clinicians and consumers.

**Objective:**

To develop theoretically driven weight loss sustaining text messages and pilot an mHealth SMS text messaging intervention to promote sustaining recent weight loss in order to understand optimal frequency and timing of message delivery, and for feasibility and usability testing. Results from the pilot study were used to design and construct a patient privacy compliant automated SMS application to deliver weight loss sustaining messages.

**Methods:**

We first conducted a pilot study in which participants (N=16) received a daily SMS text message for one month following a structured weight loss program. Messages were developed from diet and exercise guidelines. Following the intervention, interviews were conducted and self-reported weight was collected via SMS text messaging.

**Results:**

All participants (N=16) were capable of sending and receiving SMS text messages. During the phone interview at 1 month post-baseline and at 3 months post-baseline, 13/14 (93%) of participants who completed the study reported their weight via SMS. At 3 months post-baseline, 79% (11/14) participants sustained or continued to lose weight. Participants (13/14, 93%) were favorable toward the messages and the majority (10/14, 71%) felt they were useful in helping them sustain weight loss. All 14 participants who completed the interview thought SMS was a favorable communication medium and was useful to receive short relevant messages promptly and directly. All participants read the messages when they knew they arrived and most (11/14, 79%) read the messages at the time of delivery. All participants felt that at least one daily message is needed to sustain weight loss behaviors and that they should be delivered in the morning. Results were then used to develop the SMS text messaging application.

**Conclusions:**

Study results demonstrated the feasibility of developing weight loss SMS text messages, and the development of an mHealth SMS text messaging application. SMS text messaging was perceived as an appropriate and accepted tool to deliver health promotion content.

## Introduction

### Background

Nearly two-thirds of the American population is overweight (body mass index, BMI>25), of which one-third has obesity (BMI>30). Obesity is associated with multiple chronic illnesses [1] and represents a significant cost to the United States health care system [2,3]. Though people are often successful at initial weight loss, only 1 in 6 people successfully sustain weight loss over 12 months [4,5]. The rate of weight gain is highest immediately after cessation of a structured weight loss program [6]. This relapse in weight regain is attributable to a failure to maintain regular physical activity [7-9], follow a low-calorie diet [7], and monitor body weight [8,10,11]. Thus, effective interventions are needed that can follow a structured weight loss program that are accessible, affordable, and use well-accepted treatment strategies that promote sustainability and maintenance of weight loss [3,12].

Mobile phone short message service (SMS) text messaging, has the potential to serve as an intervention medium to promote sustainability of weight loss that can be easily and affordably used by clinicians and consumers. Due to widespread use and low cost of SMS text messaging, this technology pervades all age groups [13-15], many cultures [16], and socioeconomic backgrounds [14,15]. This technology allows reach across geographic boundaries and reaches people directly where they are located. Thus, it is increasingly accepted and used as a mode for health behavior change interventions rather than mediums such as the Internet or traditional telephone [17]. As of 2011, 83% of American’s own a cell phone and over 73% of those use SMS text messaging [18]. Thus, no other medium exists that can reach people as quickly and personally as SMS text messaging.

Preliminary studies suggested that SMS text messaging is an affordable mobile health (mHealth) intervention tool, and has positive short-term behavioral and clinical outcomes when compared to usual care [17,19-30] and that once a day may be appropriate to help motivate people to engage in weight loss behaviors without creating substantial burden [31]. However, a review of the literature [31] indicated that SMS text messaging as an intervention medium for weight loss is in its infancy. Furthermore, a recent study by Shapiro et al [32] showed that weight loss text messages had no effect on weight loss over 12 months but had positive effects on weight loss behaviors (eg, adequate exercise and a healthy diet) over 12 months. Though frequency and timing of messages for weight loss initiation is reported, optimal frequency and timing of following recent weight loss is still unknown.

Therefore, an intervention using text messaging was developed to help motivate people to continue their weight loss behaviors within the first month of completing a structured weight loss program. The goal was to design an intervention that would help people to stay motivated and prevent weight regain. However, 3 important aspects need to be researched effectively before we can use text messaging as a tool for sustaining weight loss. These aspects include content, frequency, and timing of the messages.

### Theoretical Frameworks

Research has demonstrated that interventions intended to change unhealthy behaviors are more likely to elicit positive changes and benefit individuals and communities if they are guided by theories of behavior change [33]. However, many theory-guided interventions that generated positive rates of initial change failed to facilitate long-term maintenance [10,34-36]. The dominant health behavior change models are successful at guiding interventions that create short-term behavior change but do little to improve sustained behavior change [36]. These models rely on the premise that the strategies people use to initiate a behavior change are the same as those used with maintenance. This is at odds with research that has demonstrated successful rates of initiation do not translate into maintenance [37,38]. Even among intervention strategies that increase the intensity or frequency of a treatment, thus delaying relapse, long-term sustainability and maintenance was not substantially improved [39-41]. Therefore, it is assumed that there are psychological differences in the processes of initiation and maintenance.

**Figure 1:**
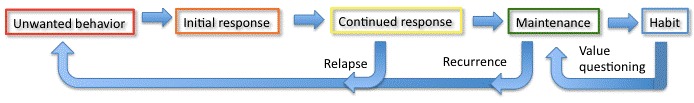
Rothman’s behavior change process [42].

To develop an intervention to focus on sustaining weight loss following initiation and completion of a structure weight loss program, Rothman’s Behavior Change Process [42] (Figure 1) was used as a guiding framework to focus specifically on continuing weight loss behaviors following an initial change. This framework focuses on transitioning from unhealthy behaviors to healthy behaviors through a 4-step process. The first step is an initial change, where for example, people with obesity begin an exercise program and healthy eating. This is followed by a sustained or continued response where people must overcome challenges and barriers to continuing the new exercise and diet behaviors. Ultimately, people move from the sustained response to a state of maintenance and then habit, where they have integrated the weight loss behaviors into their daily lives. However, for complex behaviors such as weight loss, the transition from continued response to maintenance is challenging [10,34,35]. People often relapse, revert back to their old behaviors and regain lost weight [4,5], particularly within the first month of a structured weight loss program [6].

Due to the obesity epidemic the United States faces, obesity reduction is in critical need of research on sustainability and maintenance strategies. However, different processes govern weight loss initiation versus sustainability [36,42-45]. Emerging research has suggested interventions target and frame messages about how people reach goals in their life, such as sustained weight loss, through either a prevention or promotion focus; this is known as regulatory focus theory [46]. This may be beneficial by motivating people to self-regulate and sustain recent behavioral changes [47] such as weight control [48]. This may be particularly useful at the continued response phase of the behavior change process. The regulatory focus theory argues that there are two distinct strategies that people use to reach a goal [46]. People either promote success (promotion) or prevent failure (prevention) [47]. Although any goal can be pursued with either a promotion or prevention focus, some goals are more compatible with an individual’s innate preference of promotion or prevention. When a goal matches a person’s innate preference for promotion or prevention, it creates a “fit” [47,49]. Messages that “fit” an individual’s regulatory focus resonate more with the individual and are more persuasive [49,50]. Motivational strength is enhanced when goals match a person’s regulatory focus [51]. Thus, when a “fit” occurs, people are better able to sustain self-regulation and behaviors that allow them to reach their goals such as physical activity, a healthy diet, and weight monitoring.

We must also identify amount, frequency, and timing of delivery of text messages. In regard to the message frequency, the habituation-tedium theory informs us that message frequency is important for message effectiveness [52]. Repeated exposures to messages lead to familiarity and increased effectiveness [52]. However, too many messages and repetition may lead to tedium, increased boredom, and become burdensome [52]. With regard to content, studies on content inform us that people need information on a healthy diet, exercise, and self-monitoring of weight loss behaviors [7-11].

**Figure 2:**
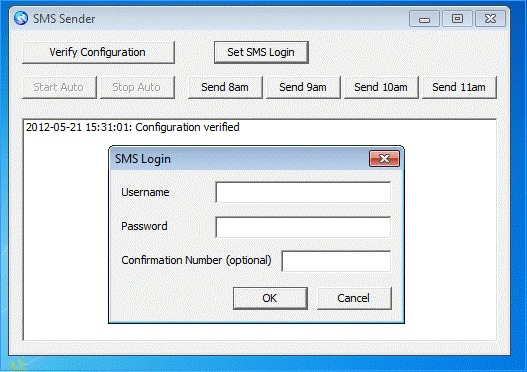
Web application for content management, powered by an SMS gateway–Manual Mode, SMS Login Configuration.

### Objectives

The objective of this feasibility and development pilot study involving the use of text messaging was to address 4 aims: (1) to develop promotion and prevention framed weight loss sustaining messages to be delivered via text messaging, (2) to assess participants’ perception of the usefulness and attitudes of the text messages, (3) to understand the frequency and timing of message delivery, and (4) to develop an automated intervention to deliver weight loss sustaining messages via SMS text messaging to people with obesity following a structure weight loss program. We will use the results from this development and pilot study for a subsequent randomized controlled trial (RCT). We chose to make an automated program so that message delivery would be consistent and standardized for all participants and could be an efficient tool for clinicians to deliver weight loss messages to ample patients any day and time of the year.

## Methods

### Message Development

Since many people begin to relapse within the first month of a structured weight loss program [6], we initially created 30 text messages, a month’s worth of daily messages on nutrition, exercise, and self-monitoring of weight, diet, and exercise. These are behaviors needed to sustain weight loss activity [7-11,53-55]. Messages were limited to 140 characters, which is less than the standard length of a single text message of 160 characters, to accommodate for limitations of different cell phone models. Message content was derived from current diet and exercise guidelines [56-58], and the Duke University Diet and Fitness Center, a residential weight management program. Nutrition messages focused on strategies to promote fullness, portion control, and to avoid binge eating and trigger foods, planning meals, coping strategies, and monitoring food intake. Exercise messages focused on benefits of exercise, physical activity goals, overcoming barriers, barriers to initiating and maintaining exercise/physical activity, monitoring exercise amount and intensity, and fitness activities. Self-monitoring messages focused on monitoring and recording of weight, food intake, exercise, identifying poor habits, and reviewing progress with weight and behavior patterns over time [59]. We then pilot tested the messages for their usefulness and relevance to people who have recently lost at least 5% of their body weight and are attempting to continue their weight loss behaviors.

We framed each message to a promotion and prevention focus for a total of 60 messages to match the Regulatory Focus Theory (see Table 1). Two expert health psychologists reviewed the messages to verify they were framed appropriately to match a prevention or promotion orientation. The messages were then pilot tested for their usefulness and relevance.

### Study Design

#### Recruitment

With IRB approval, we recruited participants from a residential weight management program in North Carolina known as the Duke Diet and Fitness Center. The Duke Diet and Fitness Center provides a residential weight management program that helps people affected by excess weight and impaired physical fitness achieve better health through weight loss, physical conditioning, and improved self-care habits. Clients enroll in a comprehensive program that provides education, practical behavioral strategies, and ongoing support to make long-term changes. The average participant who attends the Duke Diet and Fitness Center loses on average 5% of their initial body weight.

Eligible participants met the following inclusion criteria: (1) ≥18 years old, (2) able to speak and read English, (3) had completed a comprehensive diet and fitness program, (4) had a BMI≥30 at the start of the diet and fitness program, (5) lost 5% of their body weight by the beginning of the study, (6) had their own mobile phone to personally receive SMS text messaging, (7) physically able to access SMS text messaging on their phone, (8) able to continue physical activity for 3 months, (9) did not have a joint replacement scheduled within 3 months, and (10) were capable of informed consent. Participants were compensated $50 for their participation at study completion.

We recruited participants during a self-management class at the Duke Diet and Fitness Center. Those who met the inclusion criteria and agreed to participate, signed an informed consent, and completed a survey including demographic information and the Regulatory Focus Questionnaire (RFQ) to measure if the participants were innately promotion or prevention focused [60].

#### Intervention

Following informed consent, we provided participants an orientation to the intervention. Participants received messages (promotion or prevention) that matched with their RFQ results of preference for promotion or prevention. Messages were set in a queue and alternated between nutrition, exercise, and monitoring. We initially tested the frequency and delivery of the text messages and sent them out daily at an arbitrary time, 9:00 AM. We kept a message delivery log and checked it daily to ensure that appropriate daily messages were delivered to participants.

At 1 and 3 months post-baseline, we sent a text message to participants requesting them to self-report their weight. We also sent an email asking participants when they would be available to take part in a phone interview. We followed-up with non-responders with a telephone call. With participants’ permission, interviews were recorded and transcribed for analysis.

#### Measures and Variables

At baseline, we obtained through self-report gender, age, education level, and work status. Height, weight, and BMI were obtained at baseline from participants’ medical record. Clinician obtained weights were not possible following completion of the residential weight loss program since participants returned home across the United States. Self-report was deemed as the practical measurement. We used the RFQ [60] to assess innate promotion or prevention preference to reach goals. This measure consists of 11 5-point Likert-type items (5 prevention, 6 promotion), which assesses the history of individuals’ success at promotion and prevention tasks over the course of their lives. Scores range from −2 to +2. Positive scores indicate greater previous success in promotion self-regulation. Negative scores indicate greater previous success in prevention self-regulation. This scale has established reliability and validity [60].

We measured feasibility and acceptability at 1 month post-baseline through a semi-structured telephone interview guided by an interview questionnaire. We asked participants how often they read the messages, if there were times when they did not read the messages, and if they encountered technical barriers to assess feasibility and fidelity of the study design and delivery of treatment [61]. We calculated responsiveness and treatment fidelity related to receipt of the messages by how useful and favorable (attitudes) participants felt about the intervention. To assess usefulness, attitudes, and acceptability, participants were asked in the interview how useful they found the text messages, whether they liked the message content, and whether they liked text messaging as an intervention tool for helping to sustain weight loss. In addition, participants were asked if anything occurred during the 90-day study period that may have affected sustaining their weight loss, such as a major life event or participation in other weight loss programs. To assess optimal frequency and timing to deliver the messages, participants were asked their preference about the frequency and timing.

####  Data Analysis

We calculated demographic variables and change in weight using descriptive statistics with SAS Version 9.3. We analyzed interview data using conventional content analysis, a data reduction technique, to look for recurring themes [62-64] using Microsoft Excel 2007. The analysis was guided by questions we asked in the interviews on perceived usefulness, attitudes [65-67], and experiences [65-67] of the intervention. The content analysis involved dividing text into segments of information and coding them [68]. Inferences were made from the codes and collapsed into themes [69]. A second researcher reviewed approximately half of the interviews. Any disagreements were resolved through discussion and consensus. All codes were agreed upon. Results on perception of the usefulness and attitudes were divided into three categories of negative, positive, and neutral perceptions. Data on the preference for frequency and timing of message delivery were summed.

**Table 1:** Weight loss sustaining text messages framed to a promotion and prevention focus.^a^

	Promotion	Prevention
1N	Small diet changes add up. Eat breakfast every day. You will eat less during the day and it will help you reach your weight loss goals.	Eat breakfast every day. It will prevent eating more during the day and gaining weight back. Small diet changes add up.
2E	Weigh yourself daily at the same time and on the same scale. This is important. Checking on your progress will help you control your weight.	Check your progress and prevent failure. Weigh yourself daily at the same time and on the same scale. This will help you control your weight.
3M	Exercise regularly. Exercise will maintain a healthy weight, blood pressure, and reduce problems with diabetes.	Prevent diabetes problems, weight gain, and worsening blood pressure. Not exercising affects the body’s ability to handle blood sugar.
4N	Sustain your weight loss. Use the “plate method”. Fill up ½ your plate with vegetables, ¼ with starch, ¼ with protein.	Avoid eating too many calories. Use the “plate method”. Fill up ½ your plate with vegetables, ¼ with starch, ¼ with protein.
5E	Check your food intake. Record everything you eat and portion sizes. Checking increases awareness of what you are eating.	Check your food intake. Avoid falling into the trap of checking only “good days”. Record everything you eat and portion sizes.
6M	Count steps to increase the amount you walk. Use your pedometer. Set a goal of adding 150 steps a day up to 5000 steps a day or 2.5 miles.	Don’t risk falling off the exercise bandwagon. Count your steps to increase the amount you walk up to 5000 steps a day or 2.5 miles.
7N	Use smaller plates. You will still clean your plate, feel satisfied, and have better portion control.	Prevent over eating. Use smaller plates to avoid filling a larger plate with extra calories.
8E	Keep track of the things that lead to unplanned and overeating.	Prevent yourself from unplanned eating. Keep track of the things that lead to unplanned and overeating.
9M	Activity burns calories and helps maintain weight. Climbing stairs, parking further away, or walking to the office add up quickly to 30 min a day.	Don’t let exercise slip away. Small activities such as climbing stairs, parking further away, or walking to the office add up quickly to 30 min a day.
10N	Eat slowly. Put fork down between bites. Check fullness level during meal. When full push your plate away. Satisfaction takes 15-20 min.	Avoid feeling full and giving in to cravings. Eat slowly. Put fork down between bites. Check fullness level. Satisfaction takes 15-20 min.
11M	Keep exercising! After 1 year, dieters who exercise maintain most of their original weight loss.	Steer clear of gaining weight back. Dieters who do not exercise maintain only half of their original weight loss.
12N	Select healthy breakfast cereals. Follow the “5 and 5” rule—5 grams fiber & 5 or less of sugar. Or try heart-healthy oatmeal with honey!	Steer clear of high sugar breakfast cereals. Follow the “5 and 5 rule” —5 grams of fiber and 5 or less grams of sugar.
13E	Monitor your progress and how you are doing. Schedule time to review your progress in your calendar.	Remember not to forget to monitor your progress. Schedule time to review your progress in your calendar.
14M	Exercise helps more than just with weight loss. It helps to decrease high blood pressure, improve diabetes, and decrease cholesterol.	Exercise helps more than just with weight loss. It helps to prevent cancer, diabetes, high blood pressure, and gaining weight back.
15N	Reward yourself. It’s OK to have a little sweet foods such as pie, cookies, and candy, and alcohol.	Prevent yourself from overindulging. It’s OK to have a little sweet foods such as pie, cookies, and candy, and alcohol.
16M	Cross train. Vary your exercise: walk, bike, elliptical, water /chair aerobics. It will help you stay motivated and have fun!	Prevent boredom, injury, and over training with your exercise. Cross train: walk, bike, elliptical, water/chair aerobics.
17N	Identify and change habits and foods that lead to binges including risky foods kept in the house such as chips or watching TV while eating.	Avoid being tempted by foods and habits that lead to binges. Don’t keep risky foods in the house such as chips or watch TV while eating.
18E	Stay on top of how you are doing. Review your monitoring forms to check for patterns. Monitor at least 2-3 times a week.	Prevent yourself from slipping. Pick 2-3 days to monitor and every so often review your monitoring forms to check for patterns.
19M	For general fitness: exercise 30 minutes daily. 8-10 exercises, 8-15 repetitions, 1-3 sets, 30-90 second rest between sets.	Don't exercise too little and stop all together: exercise 30 minutes daily. 8-10 exercises, 8-15 repetitions, 1-3 sets, 30-90 second rest between sets.
20N	Use a grocery list for grocery shopping and only buy planned for items. This will help you buy good healthy foods.	Prevent temptation to buy unhealthy foods. Avoid meal planning or shopping for groceries when you are hungry.
21E	Limit size of portions at mealtimes by measuring planned servings. Keep measuring utensils readily available.	Don’t let portion sizes increase in size. Keep measuring utensils readily available and measure planned servings at mealtimes.
22E	Weigh yourself daily. Place the weight on a graph to see trends over time. It is natural to fluctuate daily due to things such as water.	Don’t feel surprised and upset when daily weighing. Place the weight on a graph to see trends over time. It is natural to fluctuate daily.
23N	Plan meals in advance to increase self-awareness—3 meals and up to 2 snacks per day going no longer than 4-5 hours between eating.	Plan meals in advance to avoid overeating and being tempted to select poor foods —3 meals, 2 snacks/day no more than 4-5 hrs between eating.
24E	Be aware of your blood pressure and monitor it. Check it when and where you can: at home, the doctor’s office, blood pressure machine at the drug store, etc.	Prevent or reduce high blood pressure. Keep track of your blood pressure at home, the doctor’s office, machine at the drug store, etc.
25M	Stay motivated! At the beginning of the week plan your exercise sessions and treat them like you would any other appointment.	Prevent becoming demotivated. At the start of the week plan exercise sessions and treat them like any other appointment.
26E	Monitor your blood sugar as prescribed and HbA1c every 3-6 months. Keep track of fluctuations and where they are to see how much they are.	Avoid large fluctuations in your blood sugar. Monitor your blood sugar as prescribed and HbA1c every 3-6 months.
27M	When you don’t feel like working out, bargain with yourself to exercise for just 10 minutes then see how you feel.	Don’t fall into the slippery slope of not feeling like exercising. Contract with yourself to exercise for just 10 min then see how you feel.
28N	Make small changes. Use trade-offs such as: I will have dessert every OTHER night or, I will only eat half of the dessert.	Avoid tempting foods. Use trade-offs such as: I will have dessert every OTHER night or, I will only eat half of the dessert.
29M	Any exercise is better than no exercise. Use a strategy to find a way to get in at least some exercise.	Don’t slip into the “I don’t have time to exercise” today excuse. Any exercise is better than no exercise.
30N	Use restaurant strategies: 2 vegetable servings, 1 caloric beverage. If calories are listed keep meals below 800.	Don’t be tempted to overeat when at restaurants: 2 vegetable servings, 1 caloric beverage, don’t arrive hungry. Keep meals below 800 calories.

^a^N=nutrition focus; E=exercise focus; M=monitoring focus

## Results

### Overview

We enrolled participants (N=16) between September and October 2010. Participants had a mean age of 52.0 (SD 15.5), a mean BMI of 38.1 (SD 7.8), a mean baseline weight of 206.8 pounds (SD 39.8), 81% (13/16) were female, 94% (15/16) were White, and 6% (1/16) was African American. As indicated by the RFQ, 63% (10/16) were innately promotion focused and 37% (6/16) were prevention focused. All participants had a college level education and had previous experience with SMS text messaging technology. Self-reported weight was received from 88% (14/16) of participants via SMS text messaging at 1 and 3 months, and these participants also participated in an individual telephone interview. Weight loss was sustained or continued for 86% (12/14) participants at 1-month post-baseline, and 79% (11/14) participants sustained or continued to lose weight at 3-months post-baseline. The mean change in weight from baseline enrollment to 1 month was −7.4 pounds (SD 8.2) and −13.7 pounds (SD 11.3) at 3 months.

### Acceptability

Participants reported from the interviews that the intervention was useful, defined as beneficial in helping them sustain weight loss, and were positive towards the text message content and SMS text messaging technology as an intervention medium. Interview themes indicated the participants perceived the intervention as a reinforcement tool, a reminder, a form of social support, and a useful way to receive short relevant messages directly to them.

#### Perception of the Usefulness of the Text Messages

The messages were perceived as useful in helping 71% (10/14) of the participants to sustain weight loss. Reinforcement and encouragement arose as a theme. One respondent said, “It really helped me. It was like having a pearl [of wisdom]. I liked that they reinforced behaviors”. Several participants said that the information helped to reinforce and remind them what behaviors they should be performing to sustain weight loss. For example, one participant stated that the messages “helped get the message home.” Participants felt that the message content was appropriate and reflected behaviors they needed to perform to sustain recent weight loss (a healthy diet, physical activity, and monitoring of progress). Twenty-one percent (3/14) of participants noted that they missed the contact of receiving the messages and were disappointed when the study ended.

Some (3/14, 21%) participants enjoyed the messages, but were not sure how much the messages helped them sustain their weight loss. These 3 participants felt that the messages needed to be more motivational and that additional elements such as supportive phone calls, a mentoring program in-person or online, and an online support program with other people who are losing weight should be added to the intervention. One participant said, “Takes more than [a text message] to lose weight.” Furthermore, one participant (7%) did not find the messages useful at all. This participant said the messages were annoying and redundant and that she did not enjoy receiving information she already knew.

#### Attitudes Towards the Text Messages

While 93% (13/14) of the participants were favorable toward the messages, 7% (1/14) was not favorable toward the messages. Participants reported enjoying and receiving “a daily boost of confidence”, and that they “always looked forward to it.” These messages were also viewed as social support. For example, one participant said, “It says there is somebody out here who cares and is reminding you don’t forget. It just starts your morning.” Another participant said, “It was like having a buddy,” while another said, “It was like someone was over my shoulder. It was like a conscience.”

#### Usefulness and Attitudes Towards SMS text messaging Technology

All participants thought that SMS text messaging was a favorable communication medium and was a useful way to receive messages promptly and directly. As noted by one participant, “I always have my cell phone on me.” Twenty-nine percent (4/14) noted SMS text messaging was useful because they were able to keep the messages on their phone and it was easy to read them multiple times. Fifty percent (7/14) of participants said that they read the messages multiple times, though it is unknown how many this entails. Twenty-nine percent (4/14) of participants said that they preferred SMS text messaging compared to other mediums such as email or telephone because it directly reaches them, is convenient, and is easy to use. However one older participant, > 65 years old, said that she preferred email over SMS text messaging. Twenty-one percent (3/14) said they forwarded and shared the messages with other people who were also trying to lose weight.

Another theme that emerged was that SMS text messaging is a good tool to deliver short relevant messages. Three participants said that they found the SMS text messaging useful because it consisted of small amounts of relevant information. One participant noted “I have the [weight loss program] material to go back and review, but what I am trying to figure out is what the most powerful tool [information] is for me.” This participant said that SMS text messaging made it easy to find the information and it came directly to her.

### Fidelity and Message Delivery

All 14 participants said they read the messages when they knew they arrived. Most participants (11/14, 79%) said that they read the messages every morning during the 30 days of message delivery. Several participants (3/14, 21%) did not read the messages every morning. Of these, one said that she was more tied to email and missed some messages coming in because her phone was on silent or she did not notice the message had arrived. Another was on vacation during part of the intervention and did not receive the messages during that time period. Another participant self-reported technical difficulties with her phone and stopped receiving messages after 2 weeks.

All participants (14/14) thought the frequency of receiving a daily message promoted weight loss sustaining behaviors. Participants felt that weight loss is a daily activity and that receiving messages less frequently would be less effective. Several participants (3/14, 21%) expressed that twice a day would still be appropriate and they indicated that a message in the morning and at night would be acceptable. All participants expressed that the best time to deliver daily messages was in the morning around 8:00 AM. This was expressed as an optimal time because it sets a precedent for the day. One participant noted that receiving a message later in the day may not be as effective because they may have already missed exercising or eaten something not on their dietary plan.

Technical issues did arise, such as a non-functioning phone in one participant and a silent mode or delay in checking the messages by two others. No participants had problems responding their self-reported weight via text message.

### Lessons Learned

This pilot study provided the following lessons learned for the development of an automated text message intervention for sustaining weight loss to be used in a future planned RCT. These included the following:

1. Participants want at least one daily message.
2. Messages should be delivered at 8:00 AM in a participant’s respective time zone.
3. To avoid splitting on some cell phones messages should not be beyond 140 characters.
4. Participants’ phone numbers are considered personal identifiable information (PII) and must be used with a secure system.

### Application Development

The Web application for content management, powered by an SMS text messaging gateway, was written in C++, using the Microsoft Visual C++ 2010 compiler. Three code libraries were used: the Microsoft Visual C++ runtime (for basic computer services), Microsoft Foundation Classes (for user interface services), and libcurl (for secure hypertext transfer protocol secure, HTTPS, data transmission services).

The application read a database of configuration information and subject information. Configuration information consisted of the text messages for each planned intervention group and the login information for the HTTPS-based SMS text messaging service. Subject information consisted of each participant’s telephone number, time zone, intervention start date, and intervention group.

The database was in the form of 5 text files on a secure file server. Three files held the list of messages for each intervention group, with one message on each line: arm1.txt, arm2.txt, and control.txt. One file held login information for the HTTPS-based SMS text messaging service, with the account name and password on separate lines, obfuscated by a simple encoding: login.txt. This file optionally held a confirmation number, where a text message confirming successful message delivery was sent. The final file held subject information in a comma-separated value (CSV) format: subjects.txt.

The application also wrote logs of its activity. The database of logs consisted of text files stored on the same secure server as the configuration and subject data. Each log file was named by the date of activity described within the file. The location of the secure file server was stored in a file, SMS text messaging.ini, placed in the same directory as the application itself. If this was not specified, the application defaulted to its own directory.

The application had two modes of use: manual and automatic. Manual mode was for initial setup, testing, and error recovery. Automatic mode was for regular use throughout the course of the trial. Manual functions included SMS text messaging service login configuration, configuration verification, and message sending. Automatic functions were configuration verification and message sending.

The SMS text messaging login configuration function allowed the user to specify the account name and password for the HTTPS-based SMS text messaging service. The user could also specify the confirmation number where a text message confirming successful message delivery was sent. Figure 1 shows this manual function.

Configuration verification read all of the configuration data and confirmed that there were no obvious errors. Configuration verification checked that the lists of text messages for each intervention group each contained 30 messages. Configuration verification checked that the SMS text messaging service login information was present. Finally, it checked that the subject information list was present and formatted correctly. Configuration verification had the option to be triggered manually through the user interface, and was done automatically every time text messages were sent, whether manually or automatically.

Because the intervention specified that each participant receive a text message at 8:00 AM local time, message sending in the application worked by processing the subjects in batches based on time zone. This processing was triggered manually by pressing a button in the user interface, or triggered automatically by setting the computer to run the application as a scheduled task, with an appropriate command-line option.

The application assumed that all subjects were in the continental United States, and therefore had 4 options for sending a batch of messages. The application named the batches using the time (specifically Eastern time zone). Thus, if a batch was sent to the Eastern Time zone at 8:00 AM, it was named “8am,” and the 8:00 AM batch sent to the Pacific time zone was named “11am.” When processing of a batch began, the application went through the following steps:

1. Verified the configuration
2. Read the subject information and gathered the batch of subjects that were in the current time zone
3. Using the subject start date and the current date, calculated which day of the intervention each subject was on, discarding subjects whose intervention had not started yet, or whose intervention was over
4. Logged in to the HTTPS-based SMS text messaging service
5. For each subject in the batch:
  - Selected the appropriate text message using the subject’s calculated intervention day and intervention group the subject was in (for a planned future RCT)
  - Sent the selected text message to the subject’s phone number
6. If the application was running in automatic mode, and the batch was the first batch of the day (8:00 AM batch), sent a confirmation text message to a confirmation phone number

If an error occurred at any step, the error was logged. Processing continued as much as was possible, including the text confirmation message in the final step. If errors prevented the sending of any messages, the confirmation text message included this information. If a failure occurred during automatic processing, we launched the application in manual mode to identify and fix any configuration errors and attempted to send the message again.

Automatic mode was configured with help from the operating system. For this trial, four Windows Scheduled Tasks were set up, one for each time zone. The tasks were configured to run daily at 8:00 AM, 9:00 AM, 10:00 AM, and 11:00 AM Each task launched the application with a command line option that instructed the application to automatically send the batch of messages for that particular time, like so:

- 8:00 AM: SMS text messaging.exe /run8
- 9:00 AM: SMS text messaging.exe /run9
- 10:00 AM: SMS text messaging.exe /run10
- 11:00 AM: SMS text messaging.exe /run11

The service was set up to “Run As” a user with access to the secure file server, which stored the configuration, subject, and log files. This allowed the service to run daily and send all necessary messages without regular manual intervention.

### Patient Privacy Considerations

Patient privacy was a major consideration in the design and development of the application. When designing IT applications such as this, data encryption must be written into the software development. Data are transmitted to participants from a secure server, which reads PII (telephone numbers) and then transmits data to a personal phone through a secure communication protocol HTTPS to a SMS text messaging gateway. HTTPS provides authentication of a third-party SMS text messaging service that this application used to transmit the text messages. This was critical to provide assurance that the application communicated precisely with the website it intended to and ensured the communicated content (eg, PII telephone numbers) were not read by any unintended third parties. mHealth interventions may often times use a third party to transmit SMS text messaging, video messages, voice messages, or other data onto an app. It is imperative that PII is limited regarding what is transmitted and that these third party companies or services also have privacy agreements.

Nevertheless, cell phones are often shared and other people can read information transmitted to participants. This is particularly true for SMS text messaging where user authentication is not available for an individual message at this time beyond having a password protected cell phone. Furthermore, SMS text messaging is fundamentally a non-encrypted communications protocol. Thus, we must be careful and aware of the sensitivity of the delivered content.

## Discussion

### Principal Findings

These results suggest that it was feasible to develop and deliver promotion and prevention framed weight loss sustaining messages via SMS text messaging to people who have recently completed a structured weight loss program and are in the continued response phase of the behavior change process. Most participants (11/16, 79%) sustained or continued to lose weight at 3 months post-baseline and had a mean change in weight from baseline enrollment to 3 months of −13.7 pounds (SD 11.3). Many participants felt that the messages helped them stay motivated. Several participants expressed that they felt they had a companion with the daily messages and that it was important to receive daily reminders. Of the 3 participants who had a neutral perception and the one who had a negative perception of the intervention, all indicated they would have preferred the messages to either be more motivational or have additional components such as a telephone element or online discussion group. All 3 continued to lose weight at month 3.

All participants were positive about the SMS text messaging delivery method. Participants expressed favorability towards SMS text messaging because messages were delivered directly to them. Most of the participants read the messages every day; however some technical issues did arise.

In regard to the message frequency, the habituation-tedium theory suggests that message frequency is important for message effectiveness [52]. Repeated exposures to messages lead to familiarity and increased effectiveness. However, too many messages and repetition may lead to tedium, increased boredom, and become burdensome. Thus, it is imperative that optimal frequency of message delivery be determined prior to testing an effectiveness trial. Previous studies report a daily text message had a positive clinical effect on behavior [24,27,70] and a review of the literature on SMS text messaging weight loss interventions indicated that once a day might be appropriate to help motivate people to engage in weight loss behaviors without creating substantial burden [31]. Results from suggest that once a day was deemed as the most appropriate message frequency and 8:00 AM as the best delivery time. However because weight loss is a continuous process, several participants indicated that receiving messages twice a day, preferably at morning and night would be beneficial. In relation to the habituation-tedium theory, more than twice a day would be considered burdensome and create tedium, while fewer than once a day would not be effective and fail to reach habituation. Thus, the ideal frequency for message effectiveness was at least once a day and no more than two times a day. SMS text messaging also emerged as an appropriate medium to collect self-report data on weight.

As stage 2 of Meaningful Use is upon us, the Center for Medicare and Medicaid Services guidelines call on providers and researchers to determine whether personally identifiable health data is secure in storage, with the goal of encouraging providers to encrypt data when it's transmitted to mobile devices. In the design of our SMS text messaging intervention, we specifically took strides to ensure that data would be stored on a secure server and would transmit data securely to a third party SMS text messaging service. When selecting a third party service it is crucial that one is used in which privacy rules are in place and data security is robust.

### Limitations

This feasibility and development pilot study was limited in sample size, and the majority of participants were from a more affluent and educated background, mostly White non-Hispanic, and not representative of the general US population. Furthermore, the study relied on self-report of weight. Because scales can vary, baseline report of weight at the Duke Diet and Fitness Center may differ from the self-report of weight that participants take at home. Nevertheless, the accuracy of self-reported weight has been demonstrated in Internet-based weight loss treatment programs and has shown to be comparable to observed weight [71]. In addition, participants who left the residential weight loss program did not return to the program for follow-up weights. Thus, the self-reporting of weight reflects a more realistic application and evaluation. Though we asked participants if they read every message, we did not inquire if participants read the words when the messages arrived. Furthermore, we did not assess the effects of the message framing in this pilot study. Despite the limitations, the results from this pilot study were useful and helped guide the development of an evidence-based and theoretically driven SMS text messaging intervention.

### Implications for Clinical Practice

SMS text messaging interventions have the potential to be easy-to-use tools that clinicians and consumers can use to help manage chronic conditions and sustain healthy behaviors. Because text messaging is such a ubiquitous medium of communication, it can allow providers to easily reach and deliver care directly to their patients. One can envision this SMS text messaging intervention being leveraged in primary care clinics and weight loss specific centers as a way to extend care affordably. Tools such as this could also easily be leveraged for many other self-managed chronic diseases such as diabetes, hypertension, and glaucoma.

### Conclusions

As a pilot study of a larger weight loss sustaining RCT intervention, results from this pilot study provide valuable insights on development of weight loss text messages, frequency, timing, and the development of an automated mHealth text messages intervention. Consistent with the literature [17,28,31], SMS text messaging was an appropriate and accepted tool to deliver health promotion content. Our next steps using the message content, information on frequency and timing, and application developed in this study, are to assess the effects of these weight loss-sustaining messages and the intervention on sustaining weight loss and preventing relapse. We will also examine the differential effects of framed weight loss messages on sustaining weight loss in a RCT at the Duke Diet and Fitness Center.

## Abbreviations

BMI: body mass index
CSV: comma-separated value
HTTPS: hypertext transfer protocol secure
PII: personal identifiable information
RCT: randomized controlled trial
RFQ: Regulatory Focus Questionnaire
SMS: short message service
